# Detection and Evaluation of Serological Biomarkers to Predict Osteoarthritis in Anterior Cruciate Ligament Transection Combined Medial Meniscectomy Rat Model

**DOI:** 10.3390/ijms221910179

**Published:** 2021-09-22

**Authors:** Nian-Cih Huang, Tsorng-Shyang Yang, Prabhakar Busa, Ching-Ling Lin, Ya-Chieh Fang, Ing-Jung Chen, Chih-Shung Wong

**Affiliations:** 1 Department of Anesthesiology, Tri-Service General Hospital, Taipei 114202, Taiwan; niancih@hotmail.com; 2 National Defense Medical Center, Graduate Institute of Medical Sciences, Taipei 114201, Taiwan; 3 Department of Anesthesiology, Cathay General Hospital, Taipei 106438, Taiwan; fallenseraph0212@gmail.com (T.-S.Y.); Prabhakar.busa01@gmail.com (P.B.); dr.jungchen@gmail.com (I.-J.C.); 4 Cathay Medical Research Institute, Cathay General Hospital, Taipei 106438, Taiwan; 5 Radioimmunoassay Laboratory, Cathay General Hospital, Taipei 106438, Taiwan; work5halfday@cgh.org.tw (C.-L.L.); jaa810@cgh.org.tw (Y.-C.F.); 6 Department of Internal Medicine, Cathay General Hospital, Taipei 106438, Taiwan

**Keywords:** osteoarthritis, biomarkers, anterior cruciate ligament transection, medial meniscectomy, vitamin-D3, C-telopeptide fragments of type II, leptin, cartilage oligomeric matrix protein, matrix metallopeptidase

## Abstract

Biomarkers are essential tools in osteoarthritis (OA) research, clinical trials, and drug development. Detecting and evaluating biomarkers in OA research can open new avenues for researching and developing new therapeutics. In the present report, we have explored the serological detection of various osteoarthritis-related biomarkers in the preclinical model of OA. In this surgical OA model, we disrupted the medial tibial cartilage’s integrity via anterior cruciate ligament transection combined with medial meniscectomy (ACLT+MMx) of a single joint of Wistar rats. The progression of OA was verified, as shown by the microscopic deterioration of cartilage and the increasing cartilage degeneration scoring from 4 to 12 weeks postsurgery. The concentration of serological biomarkers was measured at two timepoints, along with the complete blood count and bone electrolytes, with biochemical analysis further conducted. The panel evaluated inflammatory biomarkers, bone/cartilage biomarkers, and lipid metabolic pathway biomarkers. In chronic OA rats, we found a significant reduction of total vitamin D3 and C-telopeptide fragments of type II (CTX-II) levels in the serum as compared to sham-operated rats. In contrast, the serological levels of adiponectin, leptin, and matrix metallopeptidase (MMP3) were significantly enhanced in chronic OA rats. The inflammatory markers, blood cell composition, and biochemical profile remained unchanged after surgery. In conclusion, we found that a preclinical model of single-joint OA with significant deterioration of the cartilage can lead to serological changes to the cartilage and metabolic-related biomarkers without alteration of the systemic blood and biochemical profile. Thus, this biomarker profile provides a new tool for diagnostic/therapeutic assessment in OA scientific research.

## 1. Introduction

Osteoarthritis (OA) is the most common type of arthritis worldwide, and it affects aged people widely [1]. According to the World Health Organization (WHO), almost 70% of aged people face OA-related complications worldwide [2,3]. OA is characterized by morphological changes in the articular cartilage and subchondral bone and inflammation at the knee joints [4,5]. The prevention and management of these complaints are ideal for OA management [6,7,8].

With OA, the pain tends to be localized to the affected joints, and motion of these joints aggravates the pain [9,10]. The goal of nonsurgical treatment modalities is to reduce the pain and restore function while delaying a total knee replacement (TKR) [11,12]. Modern-day research has made it possible to manage OA by administering effective therapeutics [12,13,14].

The preclinical models of OA make it possible to study the pathophysiological changes and disease progression in OA [15]. OA is induced by various methods including anterior cruciate ligament transection (ACLT), medial meniscectomy (MMx), medial meniscus tear, ovariectomy, and combinational models. Among all combinational models, the anterior cruciate ligament transection combined with the medial meniscectomy (ACLT+MMx) model is preferred due to its reproducibility and rapid effect on OA progression [16,17,18,19]. The fast induction of OA helps in studying the effectiveness of drugs earlier than other reported OA models [20]. According to the existing literature, the severity levels of OA are enhanced with time in an incremental fashion in a combinational model [17,21].

A biomarker is a substance that can be determined in the body to predict the occurrence or development of disease [22,23]. Biomarkers are essential tools to predict and develop new therapeutics for OA [24,25]. Many biomarkers have been extensively studied, such as cartilage oligomeric matrix protein (COMP), matrix metallopeptidase (MMP3), C-telopeptide fragments of type II (CTX-II), and inflammatory markers (IL-1β, TNF-α, IL-6, transforming growth factor-beta 1 (TGFβ1), and C-reactive protein (CRP)), to predict pathophysiological changes of the cartilage and OA disease progression [26,27,28,29]. 

In this study, OA was induced by a combinational (ACLT+MMx) model. After surgery, we quantified the osteoarthritis-related biomarkers in the serum along with the complete blood count, biochemical analysis, and bone electrolytes in the blood. In addition, we further verified the morphological changes in the cartilage along with carrying out histological scoring to determine the OA severity in a time-dependent manner. 

## 2. Results

### 2.1. ACLT+MMx Induced Changes in Tibial Cartilage Morphology

**Figure 1:**
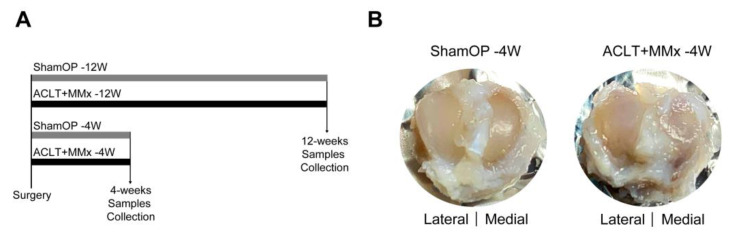
Experimental design and morphological changes of tibial cartilage. (**A**) The experimental design. (**B**) Microscopic images representing postsurgery of anterior cruciate ligament transection combined with medial meniscectomy (ACLT+MMx) method, with changes seen to tibial cartilage morphology compared with sham group (sham-OP) at the fourth week.

Figure 1A illustrates the timeline for OA induction and sample collection in Wistar rats. After surgery, the morphology of the tibial cartilage was analyzed at the 4th and 12th weeks. The blood and serum were collected for biomarker, complete blood count (CBC), and biochemical profiling. As shown in Figure 1B, ACLT+MMx surgery induced a distinct medial tibial cartilage macromorphology by week four when compared to sham-operated (sham-OP) animals. An increase in the roughness of the medial tibial cartilage implied localized deterioration with a substantial increase in the adjacent connective tissue. By contrast, the surface of the lateral tibial cartilage of the ACLT+MMx rats and the tibial cartilage in the sham-OP rats appeared smooth and bright. Thus, this macroscopic change validated the specificity of our surgery for targeting the ACLT and removing the medial meniscus.

**Figure 2:**
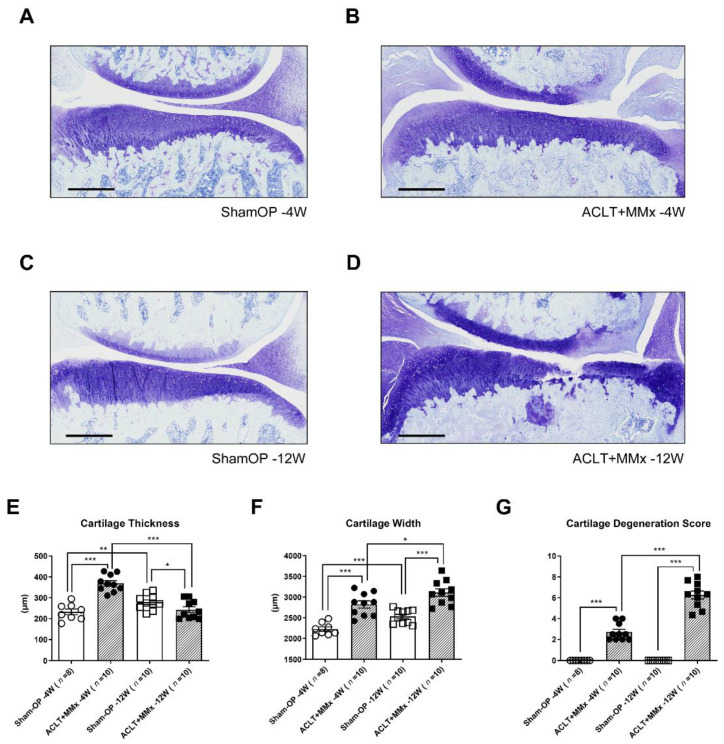
Histological changes to medial tibial cartilage postACLT+MMx surgery. Histological representation of toluidine staining tissue sections of medial tibial cartilage (scale bar = 500 μm): (**A**,**C**), sham-OP (4th and 12th weeks); (**B**,**D**), ACLT+MMx postsurgery (4th and 12th week). The mean tibial cartilage (**E**) thickness and (**F**) width, and (**G**) mean cartilage degeneration score of the three zones (outside, middle, and inside) of the medial tibial cartilage. Values are expressed as the mean ± SEM, and a *t*-test was used to analyze the data (* *p* < 0.05, ** *p* < 0.01, *** *p* < 0.001).

Furthermore, the knee joint tissues were collected on the 4th and 12th week to perform toluidine staining and verify the chondrocyte, matrix loss, and medial meniscus removal compared to the sham group [30]. As shown in Figure 2B, by the 4th week of OA induction, complete loss of the medial meniscus with a partial injury to the medial femoral cartilage was observed. In addition, there was pannus-like tissue covering the femoral and tibial cartilages’ inner and outer surfaces, implying hyperplasia of the connective tissue. Moreover, noticeable hyperplasia was observed in chondrocytes at the transitional and radial cartilage areas on the fourth week compared to the sham group. Twelve weeks postsurgery, there was an enhancement in the impact of damage seen from the fourth week. There was a substantial increase in chondrocyte hyperplasia in the inner and outer cartilage with a decaying chondrocyte morphology, along with the loss of the cartilage matrix and bone structure underneath (Figure 2D).

Further, we analyzed the medial tibial cartilage thickness, width, and cartilage degeneration score at the 4th and 12th weeks after OA induction using the ACLT+MMx method. By the fourth week, there was a noticeable increase in tibial cartilage thickness compared with the sham group as a response to early surgical OA. By contrast, there was a reduction in the tibial cartilage thickness by the 12th week, which could be attributed to the significant deterioration and erosion of the tibial cartilage (Figure 2E). Moreover, in the ACLT+MMx rat, the tibial cartilage width and degeneration score increased significantly from the 4th week to the 12th week postsurgery (Figure 2F,G). Thus, the higher cartilage degeneration score of 12th week animals confirmed this model’s chronic instability and continued OA progression. 

### 2.2. Blood and Biochemical Composition of ACLT+MMx-Induced OA Rats 

Next, the complete blood count (CBC), metabolic profile, and bone-related electrolytes were analyzed in sham-OP and OA rats. At 12 weeks postsurgery, blood samples collected from both groups showed a nonsignificant change in the CBC, glucose, total protein, triglycerides (TG), high-density lipoprotein (HDL), low-density lipoprotein (LDL), calcium, phosphorus, and magnesium levels (Table 1). Taken together, the data suggested a lack of effect in both groups at the systemic level of the blood and biochemical profiles.

### 2.3. Serum Level OA Biomarkers’ Detection and Evaluation

The detection of biomarkers is an essential tool for understating the OA disease state and progression. Therefore, the levels of proinflammatory markers were determined on the 12th week postOA induction. As shown in Table 2, proinflammatory cytokines such as interleukin 6 (IL-6), interleukin 1 beta (IL-1β), and tumor necrosis factor alpha (TNF-α) did not range in the detection levels in the 12th week for either the OA or sham group. In addition, the lack of a significant difference between the OA and sham-OP rats, regarding the transforming growth factor-beta 1 (TGf-β1) and C-reactive protein levels, indicated that our OA induction on the single joint did not involve systemic inflammation.

Furthermore, bone and cartilage metabolism biomarkers such as Vitamin D, CTX-II, PINP [31], MMP3, and COMP [32], along with lipid markers such as adiponectin [33], leptin [34], resistin, and visfatin [35], were analyzed in both groups. Among all the biomarkers studied, there was a significant increase in the MMP3 level and decreased vitamin D levels in OA rats compared to the sham group at 12 weeks postOA. By contrast, there was a significant decrease in the CTX-II level at the 12th week postsurgery in the OA group compared with the sham-OP group. In addition, there were increased adiponectin and leptin levels at the 12th week postsurgery. However, there was a decrease in the leptin level during the 4th week postOA. Finally, there was no significant change in the resistin level and an undetectable level of visfatin was found in both groups.

**Figure 3:**
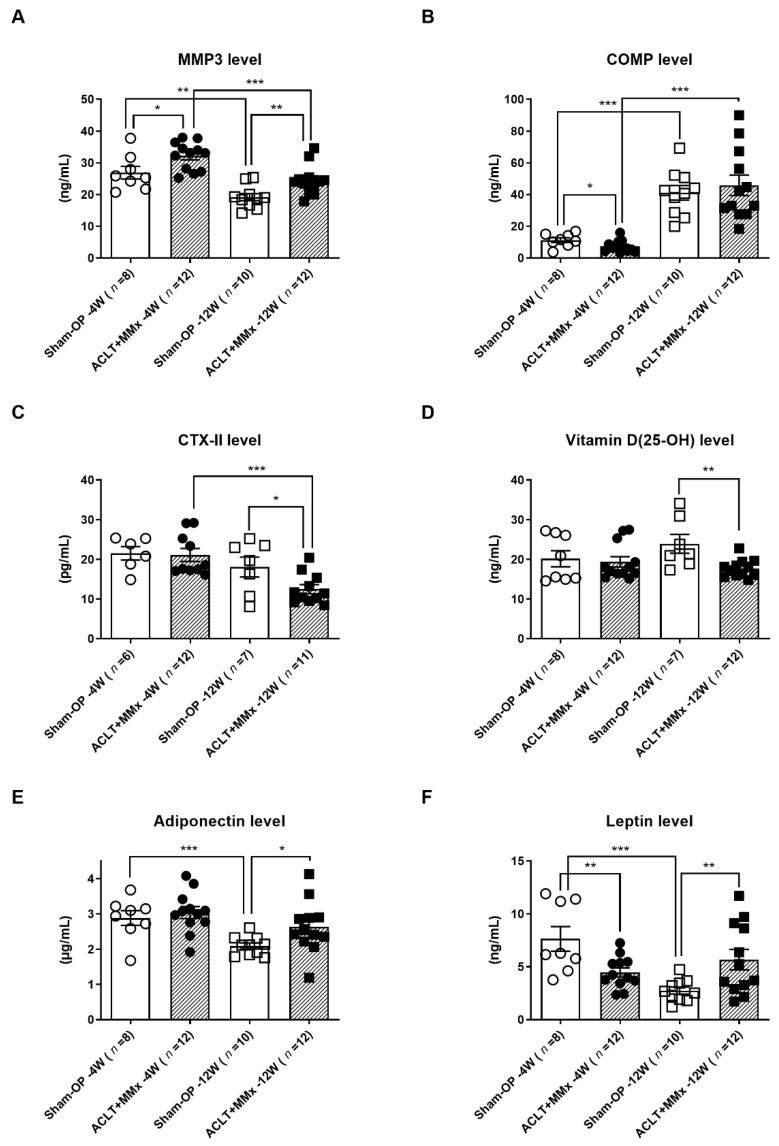
Multiple comparisons of biomarkers’ serological changes in sham and OA-induced (ACLT +MMx) rats at the 4th and 12th weeks. (**A**) MMP3, (**B**) COMP, (**C**) CTX-II, (**D**) Vitamin D (25-OH), (**E**) Adiponectin, and (**F**) Leptin. Values are expressed as the mean ± SEM, and a *t*-test was used to analyze the data (* *p* < 0.05, ** *p* < 0.01, and *** *p* < 0.001).

As shown in Figure 3, we performed multiple comparisons of the different stages and operations, finding that sham-operated rats’ aging process decreased the MMP3 (Figure 3A), adiponectin (Figure 3E), and leptin (Figure 3F), but increased the COMP (Figure 3B) level. Thus, the adiponectin, leptin, CTX-II (Figure 3C), Vitamin D (Figure 3D), and COMP of ACLT+MMx rats were significantly different when compared to the sham-operated control of the same age (4th or 12th week), and only MMP3 and leptin presented significant changes at both timepoints.

## 3. Discussion

OA is one of the most common health conditions worldwide and is associated with age-related deterioration [36,37,38]. Therefore, early detection and diagnosis of OA are essential for its proper management [39,40,41]. In this study, the combinational ACLT+MMx method used for OA induction and the biomarkers in the serum were analyzed. Further, the morphological changes of the tibial cartilage and histological changes were observed in a time-dependent manner. These findings show promising evidence for earlier diagnosis of OA. The earlier detection of OA through biomarkers can lead to effective OA treatment [26,42,43].

In this study, the tibial cartilage morphology continued to change four weeks postOA induction with the ACLT+MMx method. Notable changes were observed, such as the formation of dense connective tissue and a macroscopic change of the cartilage tissue when compared with the sham operation, confirming the successful establishment of OA [25,44,45,46]. 

Furthermore, histological studies confirmed the progression of OA with time. Compared to the sham group, the toluidine blue staining revealed more significant cartilage damage, hyperplasia, and chondrocyte degeneration with time [47,48]. Additionally, the thickness, width, and cartilage degradation scores were analyzed, finding a reduction in the cartilage thickness, an increased width of tibial cartilage, and a significant degeneration score. Those reflected the continued progression over time in OA rats compared to the healthy sham group.

The biomarkers’ detection in the biological fluids was further examined over time and compared with the findings of previous reports [49,50,51,52,53]. In contrast to the previous report [54,55], the proinflammatory markers were under the detection limit in all groups. This may be attributed to the single-joint combinational ACLT+MMx model, which could not develop meaningful systemic inflammation. The levels of TGF-1 and CRP showed no differences in the OA group compared to the sham group over time. Nonetheless, we found elevated MMP3, COMP, and adiponectin levels and lower CTX-11, vitamin D (25-OH), and leptin levels in the OA group compared to the sham group with time. In this study, the biomarkers mentioned above, derived from the cartilage extracellular matrix, could have been involved in the OA disease progression and development [26]. The results strongly support and address the value of those components, as reported in previous studies [56,57].

However, MMPs play an essential role in OA progression. Other studies have shown that MMP3 inhibitors can be used to overcome the preclinical model of OA [58]. Furthermore, MMP3 inhibitors reduce the serum levels and recover the cartilage damage with time in OA. In other studies, notable amounts of MMP3 biomarkers were detected in the serum and synovial fluid in OA [34,59]. These study results match previously reported studies, and MMP3 could thus serve as a potential biomarker in OA research for disease monitoring and therapeutic targeting. Thus, increasing evidence supports MMP3 as an attractive target for developing OA therapeutics [60,61,62,63].

Dong and his group reported the enhanced leptin levels and decreased adiponectin levels in the serum and synovial fluid in OA with metabolic syndrome, as compared to OA without metabolic syndrome [64]. It is worth noting that our animals were fed with a laboratory chow diet, but the ACLT+MMx rats still developed a significant serological alteration of adiponectin and leptin as compared to the sham-OP rats. This finding suggests a direct impact of the metabolic/lipid pathway derived from the OA of a single joint. Further exploratory experiments are needed to explain this relationship between adiponectin/leptin and the inflamed OA joint.

## 4. Materials and Methods 

### 4.1. ACLT+MMx-Induced OA Animal Model

OA induction was carried out by following the previous reports [16,17,18,19]. The animal experiments were conducted according to institutional and international standards of ethics (Principles of Laboratory Animal Care, National Institutes of Health) and approved by the Institutional Animal Care and Use Committee (IACUC) of Cathay General Hospital (Taipei, Taiwan). Adult male Wistar rats were purchased from BioLASCO Taiwan Co., Ltd. (Yilan, Taiwan) and housed in the Cathay Medical Research Center with free access to a standard chow diet and water, living with a 12-h light/dark cycle, a temperature of 22 ± 2 °C, and at 55% humidity.

ACLT+MMx surgeries were performed by a single expert on the right knee of the rats. The male Wistar rats (330–350 g) were anesthetized with 5% isoflurane. The right knee joint was shaved and sterilized using povidone-iodine solution. The medial aspect of the joint capsule was cut to open the joint. With the ACL visualized, the ACL was transected using a scalpel, and the medial meniscus was removed completely using a tenotomy scissor. Then, the joints were washed with normal saline, and the joint capsule closed with sutures. The wound area was sterilized with povidone-iodine solution, and cefazolin (100 mg/kg/day) was administered intramuscularly for three days to prevent wound infection. For the sham-group rats, the same surgical procedure was repeated without ACLT or MMx.

### 4.2. Histological Examination of the Operated Knee Joint

Rats were sacrificed under deep anesthesia at either the 4th or 12th week postsurgery. The knee joints were removed and stored in 10% formalin for 48 hours. Decalcification was performed using a decalcifying solution with ethylenediaminetetraacetic acid (EDTA) disodium (12.5%, pH 7) for four weeks. After decalcification, the joints were embedded in paraffin blocks and the slides for histological coronal sections (5 μm thick serial sections with a slide interval of 200 μm) were prepared. Toluidine blue/fast-green staining was used to examine the histological changes. The stained sections were scanned using a Slide Scanner ZEISS Axio Scan Z1 image system (Jena, Germany). The mean cartilage degeneration score (determined using the modified Osteoarthritis Research Society International scoring system) [16,17,18,19] and the mean cartilage thickness on the medial tibial plateau were evaluated using ZEN lite 3.2 software (Carl Zeiss Microscopy GmbH, Jena, Germany).

### 4.3. Complete Blood Count and Biochemical Assays

Under anesthesia, the rat’s abdomen was opened, and the blood was collected from the abdominal vein using a 5cc 21G needle. The whole blood was transferred into BD (EDTA) vacutainer tubes, which were placed in an automatic mixer for five minutes. The whole blood samples were immediately transported to NARLabs (National Laboratory Animal Center, Taipei, Taiwan) for complete blood count (CBC) analysis using a Bayer ADVIA 2120. The serum was separated by centrifugation at 2000G for five minutes and stored at −80 °C until analysis.

The biochemical assays (glucose, total cholesterol, TG, HDL, LDL, calcium, phosphorus, and magnesium) were conducted at NARLabs using a Hitachi-7080 (Holiston, MA, USA).

### 4.4. Enzyme-Linked Immunosorbent Assay (ELISA) and ElectroChemiluminescence Binding Assay

Commercially available ELISA kits were purchased for the measurement of the serological levels of biomarkers. A Nampt (Vsfatin/PBEF) ELISA kit (Cat: AG-45A-0007YEK-KI01, detection limit: 50 pg/mL) was purchased from AdipoGen Corporation (San Diego, CA, USA). A Rat/Mouse PINP EIA kit (Cat: AC-33F1, detection limit: 0.33 ng/mL) was purchased from Immunodiagnostic Systems (Tyne & Wear, United Kingdom). A Rat COMP ELISA kit (Cat: NBP2-82142, detection limit: 0.94 ng/mL) was purchased from Novus Biologicals (Centennial, CO, USA). A Rat Resistin ELISA kit was purchased from BioVendor (Brno, Czech Republic). TNF-α (Cat: 438207, detection limit: 4.3 pg/mL) and IL-6 (Cat: 437107, detection limit: 5.3 pg/mL) ELISA kits were purchased from BioLegend (San Diego, CA, USA). Rat MMP3 (Cat: ab270216, detection limit: 33.36 pg/mL), rat leptin (Cat: ab229891, detection limit: 24 pg/mL), rat adiponectin (Cat: ab239421, detection limit: 3 pg/mL), rat CRP (Cat: ab256398, detection limit 3.9 pg/mL), rat TGF-beta 1 (Cat: ab119558, detection limit: 7.8 pg/mL), and rat IL-1 beta (Cat: ab255730, detection limit: 26.58 pg/mL) ELISA kits were purchased from Abcam (Cambridge, MA, USA). All measurements were taken in duplicate or triplicate according to the manufacturer’s instructions. A standard curve was calculated for the interpolation of the concentrations of each independent serum sample. 

The total 25-hydroxyvitamin D was measured in the radioimmunoassay laboratory (Cathay General Hospital) using Cobas E411 analyzers and an Elecsys Vitamin D total II reagent kit (Roche Diagnostics GmbH, Mannheim, Germany). 

### 4.5. Statistical Analysis

The data are expressed as the mean ± SEM. All figures were plotted by GraphPad Prism version 6.01 (San Diego, CA, USA), and the multiple t-tests were employed to analyze the statistical significance difference between groups.

## 5. Conclusions

In summary, we found that a preclinical model of single-joint OA with significant deterioration of the cartilage can lead to serological changes of those biomarkers derived from the cartilage extracellular matrix, lipid metabolism, and vitamin D, but without alteration of the systemic blood and biochemical profile. Thus, the presented biomarker profile provides a new diagnostic/therapeutic assessment tool in scientific research and for medicinal products for OA disease.

## Figures and Tables

**Table 1:** Total blood composition and biochemical profile of sham-OP and ACLT+MMx-induced osteoarthritis (OA) rats (12th week).

	Sham-OP-12W	ACLT+MMx-12W
**Complete Blood Count (CBC)**		
WBC (10^3^ cells/µL)	6.59 ± 0.44	5.42 ± 0.35
RBC (10^6^ cells/µL)	8.17 ± 0.12	8.32 ± 0.12
HGB (g/dL)	13.83 ± 0.18	13.92 ± 0.11
HCT (%)	41.40 ± 0.52	41.28 ± 0.37
PLT (10^3^ cells/µL)	1189 ± 32	1066 ± 28
**Metabolic Profile**		
Glucose (mg/dL)	159.70 ± 3.62	160.52 ± 5.96
Total Cholesterol (mg/dL)	85.41 ± 3.05	83.26 ± 5.51
TG (mg/dL)	58.98 ± 7.35	66.61 ± 8.09
HDL (mg/dL)	48.39 ± 2.10	47.83 ± 4.11
LDL (mg/dL)	20.53 ± 1.66	16.83 ± 1.67
**Bone Electrolytes**		
Calcium (mg/dL)	10.43 ± 0.01	10.68 ± 0.01
Phosphorus (mg/dL)	6.60 ± 0.13	6.28 ± 0.12
Magnesium (mg/dL)	2.26 ± 0.02	2.19 ± 0.04

Blood serum was collected from independent Wistar rats at the 12th week postsurgery (sham-OP *n* = 7–10, ACLT+MMx *n* = 10–12), and data are expressed as mean ± SEM (WBC = white blood cells, RBC = red blood cells, HGB = hemoglobin, HCT = hematocrit, PLT = platelet count, TG = triglycerides, HDL = high-density lipoprotein, and LDL = low-density lipoprotein).

**Table 2:** Detection of the serological level of the biomarker at 4th and 12th weeks post-surgery.

	Sham-OP-4W	ACLT+MMx-4W	Sham-OP-12W	ACLT+MMx-12W
**Inflammatory Marker**				
IL-1β (pg/mL)	ND	ND	Below Detection Limit	Below Detection Limit
TNF-α (pg/mL)	ND	ND	Below Detection Limit	Below Detection Limit
IL-6 (pg/mL)	ND	ND	Below Detection Limit	Below Detection Limit
TGF-β1 (ng/mL)	ND	ND	96.13 ± 14.47	82.70 ± 7.80
C-Reactive Protein (μg/mL)	ND	ND	333.71 ± 13.27	353.86 ± 12.60
**Bone and Cartilage Marker**				
Vitamin D (25-OH) (ng/mL)	20.17 ± 2.03	19.38 ± 1.32	23.92 ± 2.38	17.69 ± 0.65 **
CTX-II (pg/mL)	21.50 ± 1.69	22.05 ± 1.92	18.08 ± 2.51	12.48 ± 1.14 *
PINP (ng/mL)	46.61 ± 4.24	53.59 ± 2.87	21.63 ± 1.87	19.51 ± 1.32
MMP3 (ng/mL)	26.91 ± 1.98	32.19 ± 1.28 *	19.20 ± 1.15	24.57 ± 1.36 **
COMP (ng/mL)	11.28 ± 1.48	7.42 ± 1.07 *	41.14 ± 4.57	45.79 ± 6.50
**Lipid Marker**				
Adiponectin (μg/mL)	2.88 ± 0.21	3.04 ± 0.17	2.09 ± 0.09	2.63 ± 0.21 *
Leptin (ng/mL)	7.64 ± 1.17	4.47 ± 0.43 **	2.95 ± 0.36	5.68 ± 0.97 **
Resistin (ng/mL)	18.93 ± 1.38	18.53 ± 0.86	18.93 ± 1.38	18.24 ± 1.05
Visfatin (ng/mL)	ND	ND	Below Detection Limit	Below Detection Limit

Asterisks correspond to statistical examination in comparison with sham by *t*-test (* *p* < 0.05, ** *p* < 0.01). Blood serum was collected from independent Wistar rats at the 4th and 12th weeks postsurgery (sham *n* = 7–10, ACLT+MMx *n* = 10–12), and the data are expressed as mean ± SEM (IL-1β = interleukin 1 beta, IL-6 = interleukin 6, TGF-β1 = transforming growth factor-beta 1, TNF-α = tumor necrosis factor alpha, CTX-II = C-telopeptide fragments of type II, PINP = procollagen type I N propeptide, MMP3 = matrix metallopeptidase 3, COMP = cartilage oligomeric matrix protein and ND = not determined).

## Data Availability

The data supporting the findings of this study are available within the article.
